# Supplementary material for: Implementation of Exome Sequencing in Clinical Practice for Neurological Disorders
Source: Genes (Basel). 2023 Mar 28;14(4):813. doi: 10.3390/genes14040813 (PMC10137364; doi:10.3390/genes14040813)
Supplement: Supplementary file 1 [file genes-14-00813-s001.zip › genes-2265012-supplementary.pdf]

Supplementary Table S1. List of variants detected in each disease.

Disease	Clinical diagnosis	Gene	Variant/s	Inheritance
**Ataxia**				
	Early-onset cerebellar ataxia	*APTX* (NM_175073.3)	c.837G>A p.(Trp279Ter)<br>c.837G>A p.(Trp279Ter)	AR
	Early-onset cerebellar ataxia	*COQ8A* (NM_020247.5)	c.642_649del p.(Ala215ArgfsTer69)<br>c.642_649del p.(Ala215ArgfsTer69)	AR
	Cerebellar ataxia	*KCND3* (NM_001378969.1)	c.1130C>T p.(Thr377Met)	AD
	Spastic ataxia	*POLR3A* (NM_007055.3)	c.1909+22G>A<br>c.2090C>T p.(Ser697Leu)	AR
	Cerebellar ataxia	*SPG7* (NM_003119.4)	c.233T>A p.(p.Leu78Ter)<br>c.233T>A p.(p.Leu78Ter)	AR
	Early-onset cerebellar ataxia	*SPG7* (NM_003119.3)	c.1529C>T p.(Ala510Val)<br>c.1529C>T p.(Ala510Val)	AR
	Ataxic syndrome and cognitive deterioration	*STUB1* (NM_005861.4)	c.823_824del p.(Leu275AspfsTer16)*	AD*/AR
	Ataxic syndrome	*TTR* (NM_000371.4)	c.424G>A p.(Val142Ile)	AD
**Spastic paraplegia**				
	Spastic paraplegia	*ABCD1* (NM_000033.4)	c.1998C>G p.(Tyr666Ter)	XLR
	Spastic paraplegia	*ABCD1* (NM_000033.4)	c.-34_17del	XLR
	Spastic paraplegia	*AP5Z1* (NM_014855.2)	c80_83delinsTGCTGTAAACTGTAACTGTAAA p.(Arg27_Ile28insLeuTer)<br>exon 2 and 3 deletion	AR
	Spastic paraplegia	*CYP7B1* (NM_004820.3)	c.1456C>T p.(Arg486Cys)<br>c.884C>A p.(Ala295Glu)	AR
	Spastic paraplegia	*CYP7B1* (NM_004820.3)	c.1490dup p.(Leu487PhefsTer11)<br>c.1490dup p.(Leu487PhefsTer11)	AR

	Spastic paraplegia	*DDHD1* (NM_001160148.1)	c.1999A>G p.(Arg667Gly)<br>c.1999A>G p.(Arg667Gly)	AR
	Spastic paraplegia	*DDHD2* (NM_015214.2)	c.1973G>C p.(Arg658His)<br>c.1978 G>A p.(Asp660His)	AR
	Spastic paraplegia	*KIF5A* (NM_004984.2)	c.95C>T p.(Pro32Leu)	AD
	Spastic paraplegia	*POLR3A* (NM_007055.3)	c.1909+22G>A<br>c.3429+1G>A	AR
	Spastic paraplegia	*POLR3A* (NM_007055.3)	c.1628A>C p.(Gln543Pro)<br>c.1909+22G>A	AR
	Spastic paraplegia	*PSEN1* (NM_000021.4)	c.1261A>G p.(Thr421Ala)	AD
	Spastic paraplegia	*SPAST* (NM_014946.3)	exon 1 deletion	AD
	Spastic paraplegia	*SPAST* (NM_014946.3)	c.1413+3_1413+6del	AD
	Spastic paraplegia	*SPAST* (NM_014946.3)	c.1142_1143del p.(Phe381TrpfsTer12)	AD
	Spastic paraplegia	*SPAST* (NM_014946.3)	c.1414-2A>C	AD
	Spastic paraplegia	*SPAST* (NM_014946.3)	c.1245+1G>C	AD
	Spastic paraplegia	*SPAST* (NM_014946.3)	c.1157A>G p.(Asn386Ser)	AD
	Spastic paraplegia	*SPAST* (NM_014946.3)	c.1688-3C>G	AD
	Spastic paraplegia	*SPAST* (NM_014946.3)	c.1493+2_1493+5	AD
	Spastic paraplegia	*SPAST* (NM_014946.3)	c.1676G>A p.(Gly559Asp)	AD
	Spastic paraplegia	*SPG7* (NM_003119.3)	c.987+1dupG<br>exons 11 to 15 deletion (MLPA)	AR
	Spastic paraplegia	*SPG7* (NM_003119.3)	c.773_774del (p.Val258GlyfsTer30)<br>c.1529C>T (p.Ala510Val)	AR
**Dystonia**				
	Early onset focal dystonia	*ACTB* (NM_001101.3)	c.547C>T p.(Arg183Trp)	AD
	Dystonia and spasticity	*GCH1* (NM_000161.2)<br>*AFG3L2* (NM_006796.2)	c.671A>G p.(Lys224Arg)<br>c.1847A>G p.(616Cys)	AD<br>AD
	Cervical dystonia	*ANO3* (NM_031418.4)	c.1528G>A (p.Glu510Lys)	AD

	Bulbar palsy with oromandibular dystonia	*AOPEP* (NM_001193329.3)	c.1036T>C p.(Trp346Arg)<br>c.1036T>C p.(Trp346Arg)	AR
	Dystonia and writers' cramp	*THAP1* (NM_018105.2)	c.19G>A p.(Ala7Thr)	AD
	Dystonia	*TOR1A* (NM_000113.3)	c.907_909del p.(Glu303del)	AD
	Blepharospasm	*SLC2A1* (NM_006516.3)	c.847C>T p.(Gln283Ter)	AD
**Parkinson**				
	Parkinson	*GBA* (NM_000157.4)	c.[754T>A;1093G>A] p.(Glu365Lys) p.(Phe252Ile)<br>c.1279G>A p.(Glu427Lys)[1]	RF
	Parkinson	*GBA* (NM_000157.4)	c.1342G>C p.(Asp448His)[2]	RF
	Parkinson	*LDLR* (NM_000527.5)	c.1845+1G>C[3]	AD/AR
	Parkinson	*LRRK2* (NM_198578.4)	c.4321C>G p.(Arg1441Gly)	AD
	Parkinson	*LRRK2* (NM_198578.4)	c.6059T>C p.(Ile2020Thr)	AD
	Parkinson	*PARK7 (*NM_007262.5)	hg38: 1: 7969345-7969404 deletion<br>hg38: 1: 7969345-7969404 deletion	AR
	Parkinson	*PRKN* (NM_004562.3)	c.155del p.(Asn53MetfsTer29)<br>exon 3 deletion (MLPA)	AR
	Parkinson, generalized dystonia and spasticity	*WDR45* (NM_00707.4)	c.235+5G>A[4]	XLD
**ID**				
	ID and epilepsy	*CSF1R* (NM_005211.4)	c.2442+1G>A	AD
	ID, DD, microcephaly and hypotonia	*NIPBL* (NM_)	c.1415A>G p.(His472Arg)	XLD/XLR
	ID and ASD	*WAC* (NM_016628.4)	c.1280_1281delCTinsGAG p.(Ser427Ter)	AD
	ID and facial dysmorphism	*KCNT2* (NM_198503.5)	c.569G>A p.(Arg190His)	AD
	ID and hydrocephalus	*FGFR1* (NM_023110.3)	c.880G>A p.(Glu294Lys)	AD
	ID and facial dysmorphism	*SOX5* (NM_006940.6)	c.637C>T p.(Arg213Ter)	AD
	ID	*POU3F3* (NM_006236.3)	c.539_578del p.(His180ProfsTer38)	AD
	ID and facial dysmorphism	*POGZ* (NM_015100.4)	c.2989C>T p.(Arg997Ter)	AD

	ID, facial dysmorphisms and body dysmorphic disorder	*CNOT3* (NM_014516.3)	c.169C>T p.(Arg57Trp)	AD
	Dysmorphism and procurement delay	*PPP2R5D* (NM_006245.4)	c.592G>A p.(Glu198Lys)	AD
	Opitz syndrome	*MID1* (NM_000381.3)	c.602_605del p.(Val201GlyfsTer11)	XLR
**ASD**				
	Psychomotor delay, ASD, absence of language and macrocephaly	*PPP2R5D* (NM_006245.4)	c.598G>A p.(Glu200Lys)	AD
	Angelman syndrome	*SHANK3* (NM_001372044.2)	c.3874G>T p.(Glu1292Ter)	AD
**Epilepsy**				
	Dravet syndrome	*SCN1A* (NM_001165963.4)	c.4300T>A p.(Trp1434Arg)	AD
	Epilepsy and developmental delay	*SCN1A* (NM_001165963.4)	c.4476+5G>A	AD
	Epilepsy and ID	*DYRK1A* (NM_001396.5)	c.665-1G>T	AD
	Epilepsy and ID	*SYNGAP1* (NM_006772.3)	c.333del p.(Lys114SerfsTer20)	AD
	Sotos syndrome	*NSD1* (NM_022455.5)	c.4411C>T p.(Arg1471Ter)	AD
**Other neurological disorders**				
	White matter lesions and cerebellar atrophy	*PDGFRB* (NM_002609.4)	c.2959C>T p.(Arg987Trp)	AD
	Cerebral cavernous malformation	*PDCD10* (NM_007217.4)	c.575dup p.(Ser193LysfsTer36)	AD
	Cerebral cavernous malformation	*CCM2* (NM_031443.4)	c.55C>T p.(Arg19Ter)	AD
